# The Relationship Between Parental Psychological Control and College Students’ Non-Suicidal Self-Injury: The Chain Mediation Roles of Relative Deprivation and Depression and the Moderating Role of Peer Relationships

**DOI:** 10.3390/bs15060729

**Published:** 2025-05-24

**Authors:** Sachula Cui, Qiang Bao

**Affiliations:** 1 College of Psychology, Inner Mongolia Normal University, Hohhot 010022, China; 2 Faculty of Psychology, Southwest University, Chongqing 400715, China; baoqiangswu@163.com

**Keywords:** parental psychological control, non-suicidal self-injury (NSSI), relative deprivation, depression, peer relationships, college students

## Abstract

Previous research has demonstrated that parental psychological control increases the likelihood of non-suicidal self-injury (NSSI) among college students. However, the underlying mechanisms of this association remain unclear. This study aims to investigate whether relative deprivation and depression serve as chain mediators in the relationship between paternal/maternal psychological control and NSSI, while also examining the moderating role of peer relationships. A total of 1158 college students (48.3% female; Mage = 19.26 years) completed assessments measuring parental psychological control, relative deprivation, depression, peer relationships, and NSSI. The results revealed that both paternal and maternal psychological control influence college students’ NSSI through two distinct pathways: the mediating role of depression, and a chain-mediating pathway involving relative deprivation followed by depression. Peer relationships moderated multiple associations, including those between paternal psychological control and NSSI, paternal psychological control and depression, relative deprivation and NSSI, and depression and NSSI. Notably, positive peer relationships helped mitigate the adverse effects stemming from paternal psychological control. However, peer relationships failed to buffer the negative impacts induced by maternal psychological control. These findings provide nuanced insights into the differential mechanisms through which paternal versus maternal psychological control influences college students’ NSSI. The results emphasize the necessity for distinct prevention and intervention strategies tailored to address the unique effects of paternal and maternal psychological control, thereby assisting practitioners in developing targeted approaches to reduce NSSI among college students.

## 1. Introduction

Non-suicidal self-injury (NSSI) represents a significant global public health concern. Defined as a complex and hazardous psychopathological behavior characterized by deliberate damage to one’s body tissue without suicidal intent, it manifests through methods such as cutting with sharp objects, burning with lighters, or head-banging against walls (Cipriano et al., 2017). Among Chinese college students, the prevalence of NSSI reaches 16.2% (Y. Chen et al., 2022). As a manifestation of maladjustment, NSSI serves as a primary risk factor for suicidal ideation and behaviors in this population (Hamza et al., 2012), underscoring the imperative to investigate its influencing factors and underlying mechanisms to promote positive youth development.

Existing research predominantly focuses on individual determinants (Kiekens et al., 2023; Christoforou et al., 2021), while largely neglecting the crucial role of familial environments. Notably, within China’s collectivist cultural context, family dynamics continue to exert substantial influence on college students’ psychological and behavioral development (Boyes et al., 2023). Consequently, a comprehensive understanding of NSSI mechanisms necessitates systematic examination of family-related factors. This perspective highlights the critical need to incorporate familial dimensions into theoretical frameworks and intervention strategies addressing NSSI among Chinese college students.

Parental psychological control (Barber, 1996), an intrusive parenting approach characterized by guilt induction, love withdrawal, anxiety instillation, and restriction of individual autonomy, demonstrates significant associations with maladjustment in college students (Hong & Cui, 2020). According to the integrated model of NSSI (Nock, 2009), NSSI represents a maladaptive coping mechanism, wherein traumatic experiences in parenting environments—particularly parental hostility and criticism (such as parental psychological control)—emerge as key risk factors for NSSI (J. Huang et al., 2022). While parental psychological control positively correlates with adolescent NSSI (J. Guo et al., 2022; Depestele et al., 2017), its mechanisms in college populations remain unclear (Fong et al., 2022). Emerging evidence suggests differential developmental impacts of paternal versus maternal parenting styles (Zhu et al., 2023). These differences may be attributed to shifting cultural role expectations. Against the backdrop of China’s rapid socioeconomic development and increased educational attainment, mothers have increasingly emphasized academic achievement and future success, whereas fathers have shown greater involvement in daily caregiving responsibilities. This emerging pattern—colloquially termed the ‘Tiger Mother and Cat Father’ phenomenon—suggests that maternal psychological control tends to be more pronounced compared to paternal practices (H. Y. Chen & Cheng, 2020). Previous research predominantly conflated paternal and maternal psychological control, failing to delineate their distinct operational patterns in relation to NSSI (Guérin-Marion et al., 2021). Addressing these research gaps, the present study aims to investigate the differential associations between paternal and maternal psychological control with college students’ NSSI, while exploring potential psychological mechanisms.

### 1.1. The Mediating Effect of Relative Deprivation

Relative deprivation (Smith et al., 2012) refers to a subjective state arising from perceived disadvantage through social comparisons with reference groups, eliciting anger and resentment. College students have been found to perceive the deprivation of fundamental rights through such comparisons, impairing psychosocial adaptation (Smith & Huo, 2014). Self-Determination Theory (Ryan & Deci, 2000) identifies autonomy as a core psychological need. Parental psychological control (e.g., guilt induction, autonomy restriction) directly conflicts with this need. Students experiencing high parental control may perceive relative deprivation when comparing themselves to peers with greater autonomy and have been linked to compensatory maladaptive behaviors like excessive gaming (B. Yang et al., 2021) or suicidal ideation (Pak & Choung, 2020). Although direct evidence linking parental control to deprivation is limited, analogous mechanisms observed in childhood maltreatment (Li et al., 2023) and adverse early experiences (Wang & Meng, 2024) support this hypothesized association. We therefore hypothesize a positive association between parental psychological control and relative deprivation among college students.

The socio-ecological diathesis–stress model (Swearer & Hymel, 2015) suggests that stressful events like parental control activate cognitive vulnerabilities, leading to maladaptive outcomes through distorted self/environment perceptions (Peng et al., 2024). Parental control exacerbates negative cognitive patterns (Zhou et al., 2024; J. Yang & Yang, 2025), with relative deprivation reinforcing perceived social disadvantage. Specifically, relative deprivation—as a negative subjective cognition—could reinforce perceptions of right deprivation and low social standing. This cognitive bias increases risks of both internalizing (e.g., depression via hostile attribution tendencies; Y. Guo et al., 2024) and externalizing problems (e.g., aggression; Tian et al., 2025). Crucially, heightened deprivation correlates with NSSI (Wei et al., 2025), suggesting its mediating role between parental control and NSSI.

### 1.2. The Mediating Effect of Depression

Depression encompasses negative affective states arising from maladaptive responses to stress (Thapar et al., 2012). According to the experiential avoidance model (Chapman et al., 2006), NSSI functions to escape aversive emotional experiences. Parental psychological control may trigger anger, self-blame, and frustration, particularly in individuals with emotional regulation deficits (e.g., alexithymia), prompting NSSI as a maladaptive coping mechanism. In this study, depressive symptoms are operationalized as PHQ-9-measured mood–cognition–behavior continuums, distinct from clinical depression diagnoses requiring DSM-5 criteria. This distinction is critical given symptom overlap across psychiatric conditions (e.g., anxiety, PTSD) in non-clinical samples.

Parental psychological control constitutes a risk factor for depression among college students, with substantial research confirming its positive association with depressive symptoms (Hwang et al., 2024; Dong et al., 2022). A meta-analysis (Chyung et al., 2022) further revealed a moderate correlation between parental psychological control and depression. Studies indicate that highly depressed individuals are more likely to employ NSSI as an emotional regulation strategy (Wu et al., 2023), and the positive relationship between depression and NSSI has been consistently supported by empirical evidence (Niu et al., 2024; Lei et al., 2024). Although preliminary evidence suggests depressive symptoms may mediate the parental control–NSSI link in adolescents (C. Liu et al., 2022), this mechanism remains unexplored in college populations. The current research examines whether depression mediates the associations between paternal and maternal psychological control and NSSI, respectively.

### 1.3. The Chain-Mediating Effect of Relative Deprivation and Depression

Perceived relative deprivation (Smith et al., 2012) is recognized as a significant factor influencing individual psychological development, exerting detrimental effects on psychological adjustment and demonstrating positive correlations with emotional disorders (J. Liu et al., 2024). Beshai et al. (2017) examined the relationship between relative deprivation and depression using four independent samples, revealing that relative deprivation impacts depression through negative self-cognitions. An analysis of large-scale Chinese sample data (J. Zhang & Tao, 2013) indicated that college students reporting higher levels of personal relative deprivation exhibit more severe depressive symptoms compared to those with lower deprivation reports. Given that parental psychological control influences NSSI in college students through cognitive and emotional pathways, it is reasonable to posit that both paternal and maternal psychological control may predict NSSI through the chain-mediating roles of relative deprivation and depression.

### 1.4. The Moderating Effect of Peer Relationships

Not all individuals exposed to parental psychological control engage in NSSI, indicating potential protective factors. Peer relationships, defined as connections established and developed through interpersonal interactions among individuals of similar ages or psychological maturity levels (Zou, 1998), constitute a key mental health determinant for college students. According to Ecological Systems Theory (Bronfenbrenner, 1979), familial and peer interactions jointly shape individual development. Empirical evidence indicates that positive peer relationships can buffer against mental health problems resulting from adverse parenting practices (Roach, 2018). Specifically, robust peer relationships demonstrate significant negative correlations with social anxiety (Spence & Rapee, 2016), depression (Jiang & Wang, 2020), and loneliness (Chiao et al., 2022). Consequently, peer relationships may serve as a moderating factor in the associations between parental psychological control and NSSI, relative deprivation, and depression.

The Stress-Buffering Model (Cohen & Mckay, 2020) posits that perceived social support enhances individuals’ stress-coping capacities. High-quality peer relationships help mitigate the adverse effects of parental control, facilitate effective management of negative emotions, and consequently reduce the need for NSSI as an emotional regulation strategy, thereby promoting psychological adaptation (Porter et al., 2021). Empirical evidence demonstrates that robust peer relationships furnish college students with social resources to modulate maladaptive emotions and foster the development of a positive self-concept, which contributes to cognitive maturation (Prinstein & Giletta, 2020). Such relationships also strengthen emotional regulation skills, alleviating the detrimental cognitive and emotional impacts of relative deprivation and depression (Zhao & Peng, 2021) and ultimately diminishing NSSI (Zhou et al., 2023). Peer relationships may serve as moderating factors in the associations between relative deprivation and depression, between relative deprivation and NSSI, and between depression and NSSI.

**Figure 1:**
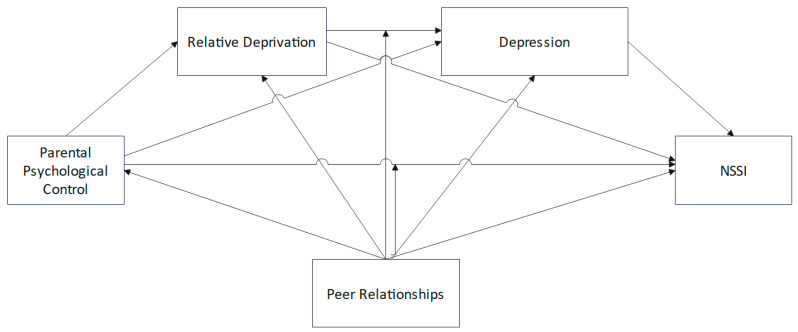
Hypothetical moderated mediation model. Note: NSSI, non-suicidal self-injury.

Building on the limitations of existing research, combined with relevant theories, this study employs a cross-sectional design to examine the relationships between paternal psychological control, maternal psychological control, and college students’ NSSI, as well as the chain-mediating effects of relative deprivation and depression and the moderating role of peer relationships. The study proposes the following hypotheses (Figure 1): H1. Paternal psychological control and maternal psychological control are positively correlated with college students’ NSSI. H2. Paternal psychological control and maternal psychological control, respectively, influence college students’ NSSI through the mediating role of relative deprivation. H3. Paternal psychological control and maternal psychological control, respectively, influence college students’ NSSI through the mediating role of depression. H4. Paternal psychological control and maternal psychological control, respectively, influence college students’ NSSI through the chain-mediating roles of relative deprivation and depression. H5. Peer relationships moderate various pathways between paternal/maternal psychological control and college students’ NSSI.

## 2. Materials and Methods

### 2.1. Participants

Participants were recruited from a Chinese university using convenience sampling. A total of 1200 questionnaires were administered electronically, and all invited participants completed the web-based survey. Following data quality screening, 42 invalid responses (e.g., patterned responding) were excluded, resulting in 1158 valid cases (96.5% retention rate). The final sample consisted of 1158 college students (48.3% female), with a mean age of 19.26 years (SD = 0.99). Among them, 15.1% reported fathers with educational attainment below junior high school, compared to 19.2% of mothers. Additionally, 30.5% of families reported a monthly household income of CNY 5000 or higher. Prior to participation, all individuals were informed of their right to withdraw and provided electronic informed consent. The study received ethical approval from the Institutional Review Board of Inner Mongolia Normal University. A notable limitation is the lack of psychiatric diagnostic data, which may confound the analysis of non-suicidal self-injury (NSSI) mechanisms discussed herein.

### 2.2. Measures

#### 2.2.1. Parental Psychological Control

The Parental Psychological Control Questionnaire was employed to measure psychological control exerted by parents on their children (Shek, 2005). The questionnaire comprises two subscales: paternal psychological control and maternal psychological control. Each subscale consists of 10 identical items differentiated by the terms “father” and “mother” in their respective contexts. Utilizing a 5-point Likert scale ranging from 1 (strongly disagree) to 5 (strongly agree), the instrument calculates a total score by summing all items, with higher aggregate scores indicating greater levels of parental psychological control. In this study, the Cronbach’s *α* reliability coefficients for paternal and maternal psychological control subscales were 0.95 and 0.96, respectively.

#### 2.2.2. Relative Deprivation

The Relative Deprivation Questionnaire was employed to measure college students’ relative deprivation (Ma, 2012). The scale comprised four items, which were rated on a 6-point Likert scale ranging from 1 (strongly disagree) to 6 (strongly agree). Greater scores are indicative of increased levels of relative deprivation. Cronbach’ s *α* values in this study were 0.77.

#### 2.2.3. Depression

The 9-item Patient Health Questionnaire (PHQ-9) (Kroenke & Spitzer, 2002) was employed to measure college students’ depressive symptoms, over a 2-week recall period. The scale comprised nine items, which were rated on a 4-point Likert scale ranging from 0 (not at all) to 3 (nearly every day), where higher total scores reflect greater symptom severity. Cronbach’ s *α* values in this study were 0.92.

#### 2.2.4. Peer Relationships

The peer relationship subscale of the Class Interpersonal Relationship Harmony Questionnaire (B. B. Chen & Li, 2009) was employed to measure college students’ peer relationships. This subscale comprises six items rated on a five-point Likert scale, with response options ranging from 1 (Never) to 5 (Always). Cronbach’ s *α* values in this study were 0.93

#### 2.2.5. Non-Suicidal Self-Injury

The Non-Suicidal Self-Injurious Questionnaire (Feng, 2008) was employed to measure college students’ NSSI. The questionnaire consists of 18 questions, each of which consists of two parts: an assessment of the number of non-suicidal self-injuries (four levels: 0, 1, 2–4, and 5 or more) and an assessment of the degree of bodily harm (five levels: none, mild, moderate, severe, and very severe). Total NSSI severity scores were calculated by multiplying frequency by physical severity ratings, with higher composite scores indicating greater self-harm intensity. Cronbach’ s *α* values in this study were 0.97.

### 2.3. Data Analyses

This study utilized SPSS 20.0 software for data analysis, including the calculation of means, standard deviations, and correlations of key variables. Model 6 in PROCESS was employed to examine the chain mediation model, while Model 92 in PROCESS was adopted to test moderating effects. Harman’s single-factor test was conducted to assess potential common method bias. The results indicated that, without rotation, nine factors exhibited eigenvalues greater than 1, with the first factor explaining 32.69% of the variance (<40%). Consequently, the data in this study demonstrated no significant common method bias.

## 3. Results

### 3.1. Descriptive Statistics

The study revealed that the NSSI rate among college students was 17.78%. As shown in Table 1, the means and standard deviations of the major variables were reported. Paternal psychological control was positively correlated with maternal psychological control, relative deprivation, depression, and NSSI, but negatively correlated with peer relationships. Maternal psychological control exhibited positive correlations with relative deprivation, depression, and NSSI, while showing a negative correlation with peer relationships. Relative deprivation was positively correlated with depression and NSSI, yet negatively correlated with peer relationships. Depression demonstrated a positive correlation with NSSI and a negative correlation with peer relationships. Peer relationships were positively correlated with NSSI.

### 3.2. Testing for Mediation Effect

**Figure 2:**
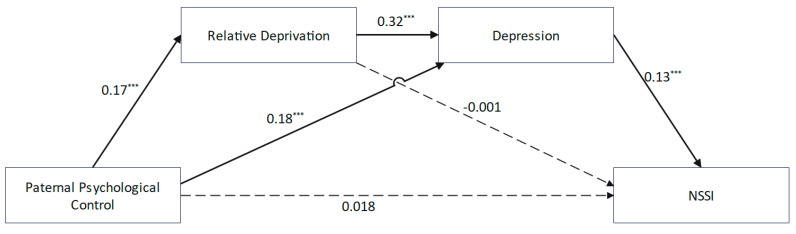
A chain-mediated model of paternal psychological control and NSSI. *** *p* < 0.001.

The chain mediation analysis was conducted using PROCESS Model 6 with standardized key variables. After controlling for gender and age, the results (Figure 2) indicated that paternal psychological control was positively correlated with NSSI (*β* = 0.05, *p* < 0.001). However, this relationship became non-significant (*β* = 0.02, *p* > 0.05) after introducing the mediating variables. Paternal psychological control showed positive correlations with relative deprivation (*β* = 0.17, *p* < 0.001) and depression (*β* = 0.18, *p* < 0.001). Relative deprivation was positively correlated with depression (*β* = 0.32, *p* < 0.001) but had no significant association with NSSI (*β* = −0.001, *p* > 0.05). Depression was significantly correlated with NSSI (*β* = 0.13, *p* < 0.001). While the direct effect of paternal psychological control on NSSI was non-significant, two indirect pathways were identified (Table 2): paternal psychological control → depression → NSSI (*β* = 0.02, *SE* = 0.005, 95% CI [0.013, 0.035]) and paternal psychological control → relative deprivation → depression → NSSI (*β* = 0.01, *SE* = 0.002, 95% CI [0.004, 0.011]).

**Figure 3:**
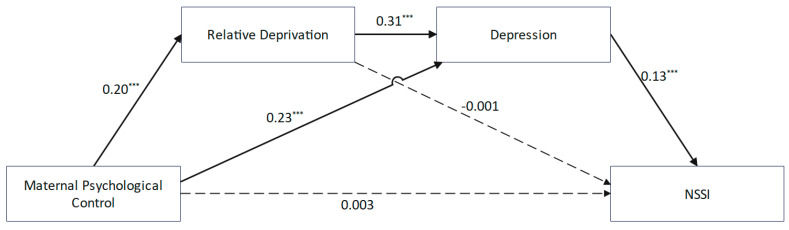
A chain-mediated model of maternal psychological control and NSSI. *** *p* < 0.001.

The results (Figure 3) demonstrated that maternal psychological control was positively correlated with non-suicidal self-injury (NSSI) (*β* = 0.04, *p* < 0.001). However, this relationship became non-significant (*β* = 0.003, *p* > 0.05) after introducing the mediating variables. Maternal psychological control exhibited positive correlations with relative deprivation (*β* = 0.20, *p* < 0.001) and depression (*β* = 0.23, *p* < 0.001). Relative deprivation was positively correlated with depression (*β* = 0.31, *p* < 0.001) but showed no significant association with NSSI (*β* = −0.001, *p* > 0.05). Depression was significantly correlated with NSSI (*β* = 0.13, *p* < 0.001). While the direct effect of maternal psychological control on NSSI was non-significant, two indirect pathways were identified (Table 3): maternal psychological control → depression → NSSI (*β* = 0.03, *SE* = 0.007, 95% CI [0.018, 0.045]) and maternal psychological control → relative deprivation → Depression → NSSI (*β* = 0.01, *SE* = 0.002, 95% CI [0.004, 0.013]).

### 3.3. Testing for Moderated Mediation

The moderating effects of peer relationships in the full model were examined using PROCESS Model 92 with standardized key variables. After controlling for gender and age, the results (Table 4) indicated that peer relationships did not moderate the relationship between paternal psychological control and relative deprivation. However, peer relationships moderated the relationships between paternal psychological control and depression and between relative deprivation and depression. Peer relationships also moderated the relationship between paternal psychological control and NSSI and between depression and NSSI. Further analysis (Table 5) revealed that in the association between maternal psychological control and NSSI, peer relationships only moderated the relationship between depression and NSSI.

**Figure 4:**
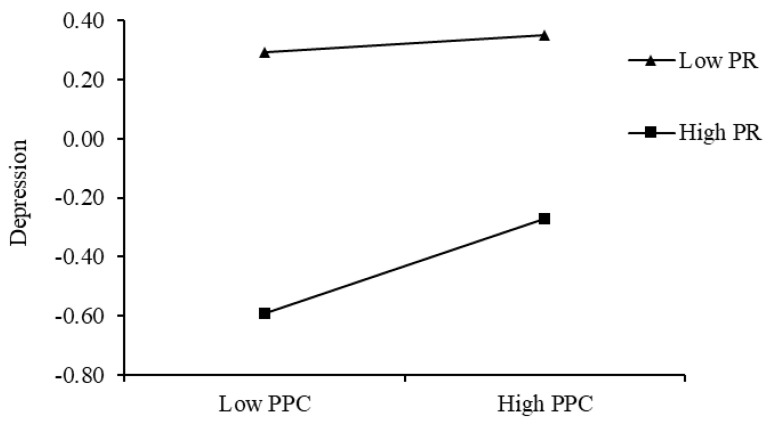
Interactive effect of paternal psychological control and peer relationships on depression.

To further examine the moderating role of peer relationships in the association between paternal psychological control and depression, peer relationships were categorized into high and low groups (±1 SD). The results (Figure 4) revealed that among college students with low peer relationships (M − 1 SD), the relationship between paternal psychological control and depression was non-significant (simple slope = 0.02, *p* > 0.05). In contrast, among students with high peer relationships (M + 1 SD), paternal psychological control was significantly positively correlated with depression (simple slope = 0.14, *p* < 0.001). The protective role of peer relationships was more pronounced under conditions of low paternal psychological control. As paternal psychological control increased, the protective effect of peer relationships gradually diminished.

**Figure 5:**
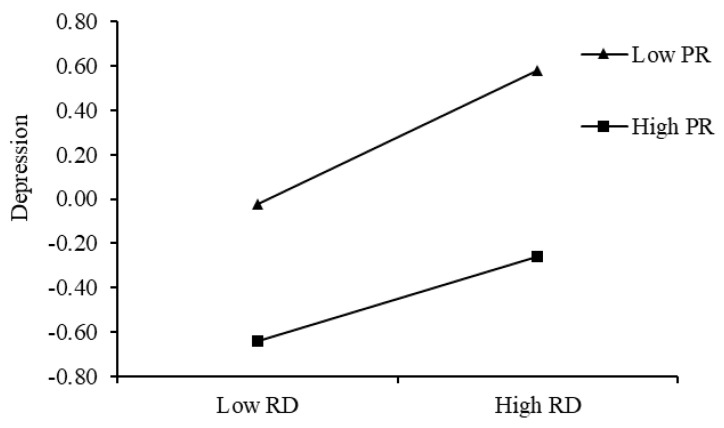
Interactive effect of relative deprivation and peer relationships on depression.

To examine the moderating role of peer relationships in the association between relative deprivation and depression, the results (Figure 5) demonstrated that among college students with high peer relationships (M + 1 SD), relative deprivation was significantly positively correlated with depression (simple slope = 0.18, *p* < 0.001). Notably, this relationship was even more pronounced among students with low peer relationships (M − 1 SD) (simple slope = 0.28, *p* < 0.001). These findings indicate that peer relationships buffered the association between relative deprivation and depression.

**Figure 6:**
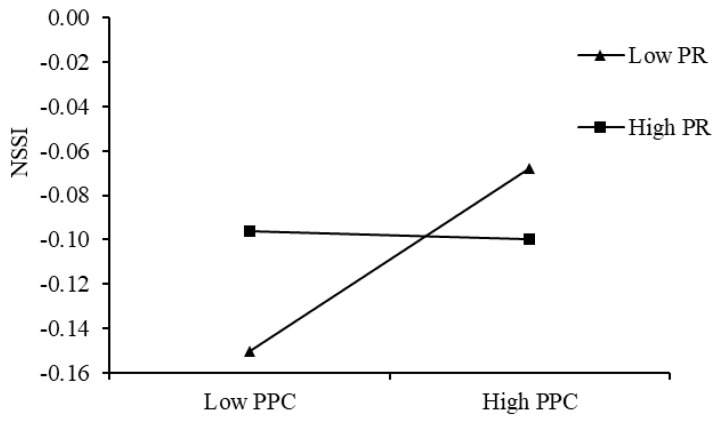
Interactive effect of paternal psychological control and peer relationships on NSSI.

To examine the moderating role of peer relationships in the association between paternal psychological control and NSSI, peer relationships were categorized into high and low groups (±1 SD). The results (Figure 6) indicated that among college students with low peer relationships (M − 1 SD), paternal psychological control was significantly positively correlated with NSSI (simple slope = 0.04, *p* < 0.01). In contrast, among students with high peer relationships (M + 1 SD), the relationship between paternal psychological control and NSSI was non-significant (simple slope = −0.003, *p* > 0.05). These findings suggest that peer relationships buffered the association between paternal psychological control and NSSI.

**Figure 7:**
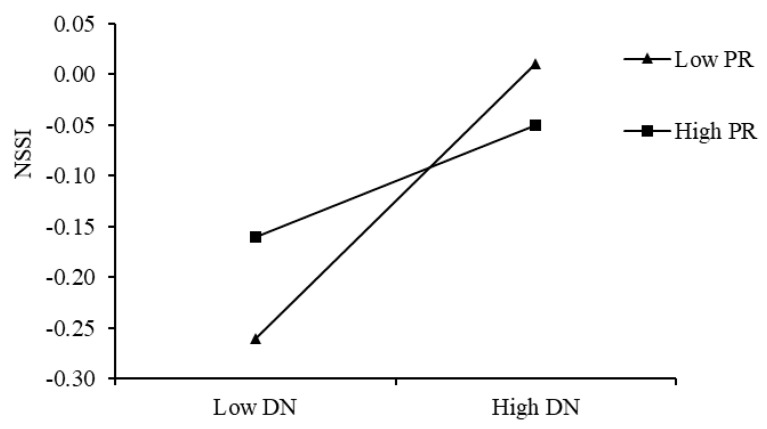
Interactive effect of depression and peer relationships on NSSI.

To examine the moderating role of peer relationships in the association between depression and NSSI, peer relationships were categorized into high and low groups (±1 SD). The results (Figure 7) demonstrated that among college students with low peer relationships (M − 1 SD), depression was significantly positively correlated with NSSI (simple slope = 0.16, *p* < 0.001). In contrast, among students with high peer relationships (M + 1 SD), the association between depression and NSSI was attenuated (simple slope = 0.07, *p* < 0.01). These findings indicate that peer relationships mitigated the relationship between depression and NSSI.

## 4. Discussion

This study constructed a moderated chain mediation model to explore the relationships between paternal and maternal psychological control and NSSI among college students, as well as the chain-mediating roles of relative deprivation and depression and the moderating role of peer relationships. The results revealed that both paternal and maternal psychological control were positively correlated with NSSI among college students, supporting Hypothesis 1. These findings align with existing research (Fong et al., 2022) and underscore that parental psychological control within the family environment remains a significant risk factor for NSSI (Flamant et al., 2020).

Self-Determination Theory (Ryan & Deci, 2000) posits three basic psychological needs: autonomy, competence, and relatedness. When these needs are fulfilled, college students achieve psychological well-being and adaptive development; conversely, chronic frustration of these needs may lead to psychological maladjustment and maladaptive behaviors. On one hand, parental psychological control restricts students’ autonomy. When unable to express autonomy healthily, students may resort to NSSI to regain a sense of bodily control. On the other hand, college students are in a transitional phase of seeking independence while remaining dependent. Parental psychological control may undermine their sense of competence, thereby reducing self-esteem (Plunkett et al., 2016), which serves as a critical protective factor against NSSI (Forrester et al., 2017).

Furthermore, these results support the Integrative Model of NSSI (Nock, 2009). Within collectivist cultural contexts, parental psychological control—particularly hostility and criticism—may diminish students’ sense of relatedness, impairing parent–child relationships. Poor parent–child relationships are a well-documented risk factor for NSSI (Martin et al., 2016).

### 4.1. The Mediating Effect of Relative Deprivation

Contrary to previous findings, this study revealed that the mediating role of relative deprivation in the relationship between parental psychological control and NSSI was non-significant. Therefore, Hypothesis 2 was not supported. According to Relative Deprivation Theory (Smith et al., 2012), relative deprivation refers to negative cognitions and emotions arising from unfavorable social comparisons, which tend to be negatively associated with mental health. Previous studies have found that family economic hardship among adolescents influences NSSI through the mediating effect of relative deprivation (Liao et al., 2024). A recent longitudinal study demonstrated that relative deprivation affects NSSI fully through the mediating role of emotional symptoms (Wei et al., 2025). Based on these prior studies, three potential explanations emerge for the non-significant mediating effect of relative deprivation in this study: First, earlier research focused on adolescents as participants, whereas this study examined college students. Compared to adolescents, college students possess more cognitive and behavioral strategies to mitigate negative emotions (e.g., problem-solving skills, social support). Additionally, adolescents’ less mature psychological development may increase their susceptibility to imitating peers’ maladaptive behaviors, amplifying the impact of relative deprivation. Second, relative deprivation has been shown to correlate more strongly with outwardly directed aggressive behaviors (e.g., bullying) among college students (Y. Guo & Xia, 2023). When faced with autonomy deprivation, college students may prioritize externalizing aggression (toward others) over internalizing aggression (NSSI). Third, the negative emotions triggered by relative deprivation fully mediated its relationship with NSSI, resulting in a non-significant direct association. This suggests that emotional dysregulation—rather than relative deprivation itself—serves as the proximal factor driving NSSI.

### 4.2. The Mediating Effect of Depression

Depression mediated the relationship between parental psychological control and NSSI, which supports Hypothesis 3 and aligns with prior findings (C. Liu et al., 2022). These results corroborate the Experiential Avoidance Model of NSSI (Chapman et al., 2006). First, NSSI functions as a maladaptive coping mechanism for college students to regulate dysphoric emotions. When confronted with excessive parental intrusion and withdrawal of affection, students may develop persistent sadness and self-criticism. To suppress or escape depressive affect, they may resort to NSSI as an emotion regulation tactic, using physical harm to alleviate psychological distress (Taylor et al., 2018). Second, NSSI may function as an interpersonal communication strategy. When students lack effective emotion regulation skills to express negative emotions appropriately, they may engage in NSSI to manage their emotional, cognitive, and social functioning. Through NSSI, individuals attempt to influence, control others, or terminate aversive interactions (Brown & Plener, 2017). Notably, depression fully mediated the association between parental psychological control and NSSI. This underscores the need for practitioners to prioritize the development of emotion regulation skills in college students and increase clinical attention to those exhibiting depressive tendencies.

### 4.3. The Chain-Mediating Effect of Relative Deprivation and Depression

Parental psychological control was associated with NSSI through sequential mediation pathways involving relative deprivation and depression, thereby supporting Hypothesis 4. These results align with prior research (Wei et al., 2025), which suggests that the association between relative deprivation and NSSI is fully accounted for by depression. Parental psychological control, characterized by restrictions on autonomy, exacerbates college students’ perceived relative deprivation of self-determination, fostering negative cognitions and emotions that ultimately culminate in depressive symptoms. To alleviate such distress, students may engage in NSSI for immediate emotional relief.

The parental psychological control → relative deprivation → depression → NSSI pathway elucidates the complex interplay among family dynamics, social cognition, and individual behavior. Under authoritarian parental control, students may observe peers enjoying greater autonomy (e.g., freedom in social relationships or leisure time), which triggers intense anger, depressive feelings, and perceptions of injustice (Karreman & Bekker, 2012). Prolonged suppression of anger and depression may erode self-esteem, leading to self-directed aggression (NSSI) (Otte et al., 2019). Within collectivist cultural contexts, families remain pivotal in shaping college students’ development. Parenting styles significantly influence students’ mental health and emotion regulation capacities (Paley & Hajal, 2022). These findings highlight the need for practitioners to prioritize interventions addressing family dynamics and enhancing emotion regulation skills to safeguard students’ psychological well-being.

### 4.4. The Moderating Effect of Peer Relationships

This study found that peer relationships served as a moderator in the association between paternal psychological control and NSSI, as well as in the associations between (a) parental psychological control and depression, (b) relative deprivation and depression, and (c) depression and NSSI. However, in the association between maternal psychological control and NSSI, this moderating effect was not observed; peer relationships only moderated the depression–NSSI link. These results suggest distinct mechanisms underlying the effects of paternal and maternal psychological control on NSSI, providing partial support for Hypothesis 5.

Poor-quality peer relationships were associated with higher depression scores. Among students with high-quality peer relationships, those exposed to low paternal psychological control exhibited the lowest depression scores, suggesting that peer relationships may provide partial protection. Notably, even students with high-quality peer relationships showed elevated depression as paternal psychological control intensified. This underscores the necessity of addressing both familial and peer environments to prevent depression among college students.

As relative deprivation increased, depression scores rose irrespective of peer relationship quality. However, the strength of relative deprivation’ s predictive effect on depression was significantly attenuated among students with high-quality peer relationships compared to those with poor-quality peer relationships. Reducing psychologically controlling parenting practices and fostering healthy peer relationships may not only mitigate students’ relative deprivation but also alleviate depression—a pattern consistent with the Stress-Buffering Model (Cohen & Mckay, 2020).

The study further revealed that high-quality peer relationships fully mitigated the risk of NSSI stemming from paternal psychological control. High-quality peer relationships foster a sense of belonging and competence in college students, enhancing their psychological resilience (Karreman & Bekker, 2012), and are associated with reduced NSSI incidence (B. Zhang et al., 2023). In contrast, students with low-quality peer relationships were more vulnerable to the adverse effects of paternal psychological control, resorting to NSSI as a maladaptive coping strategy. Close peer relationships provide acceptance, understanding, and companionship, which enable individuals to vent negative emotions while alleviating loneliness and helplessness (L. Huang et al., 2021). This emotional support not only reduces depression risk but also facilitates disengagement from dysphoric states (Abraham & Kerns, 2013), thereby diminishing depression-driven NSSI. Collectively, these findings underscore that peer relationships play a critical role in preventing NSSI among college students.

Notably, the findings of this study suggest that, for Chinese college students, familial environments—particularly the enduring negative influence of mothers—remain profoundly impactful. Peer relationships did not demonstrate significant buffering effects in the associations between maternal psychological control and depression, deprivation, or NSSI. This implies that, within collectivist cultural frameworks such as China, the influence of maternal parenting styles may persist across an individual’s developmental trajectory. This phenomenon may originate from traditional Chinese filial piety (Bedford & Yeh, 2019). Specifically, when college students perceive themselves as failing to meet maternal expectations in academic and career domains, they may develop maladaptive self-criticism. Over time, such persistent self-criticism could manifest as NSSI, potentially serving as an emotion regulation strategy. Attachment theory (Bowlby et al., 1992) posits that mothers, as primary attachment figures, exert far greater influence than peers. When maternal psychological control operates as a chronic stressor, the protective capacity of peer support becomes constrained.

To address mental health concerns among college students, interventions should prioritize family-related factors, particularly the detrimental effects of parental maltreatment on NSSI (Chia et al., 2025). Additionally, peer relationships may serve as a potential buffer against the link between depression and NSSI; thus, their role should be systematically incorporated into intervention frameworks. Although peer relationships cannot alleviate depression stemming from maternal psychological control, they may still attenuate depression-driven NSSI to some extent.

### 4.5. Limitations and Future Research

The current study has several limitations. First, the cross-sectional design limits causal inference, necessitating the use of longitudinal designs in future studies. Second, the exclusive reliance on self-reported data introduces potential common method bias; future research should implement multi-informant assessments (e.g., parental or teacher reports) to address this issue. Third, participants were sampled from a single geographic region, which constrains generalizability. Subsequent work should enhance sample diversity through inclusion of individuals representing varied socioeconomic and cultural backgrounds. Fourth, critically, given that this study was conducted within a collectivist cultural context, cross-cultural comparisons are essential to examine the mechanisms underlying parental psychological control and NSSI across different cultural frameworks. Furthermore, the omission of controlling for family socioeconomic status (SES) and parental educational attainment in the analysis may introduce residual confounding, thereby biasing the estimated relationship between parental psychological control and NSSI.

This study did not utilize structured clinical interviews to assess participants’ psychiatric diagnoses, limiting the ability to rigorously exclude comorbid mental disorders (e.g., anxiety disorders, borderline personality disorder) that may confound NSSI. Although tools like the PHQ-9 are well validated for screening depressive symptom severity, discrepancies between self-reported symptoms and formal diagnostic criteria could introduce measurement bias in mediation analyses. Future research should combine clinical diagnostic assessments with dimensional symptom measures (e.g., transdiagnostic traits) to better elucidate the psychological mechanisms underlying NSSI.

## 5. Conclusions

This study developed a moderated chain mediation model to examine the relationships between paternal/maternal psychological control and non-suicidal self-injury (NSSI) among college students, along with the mediating effects of relative deprivation and depression and the moderating role of peer relationships. The results indicated direct positive associations between both paternal and maternal psychological control and NSSI. Although relative deprivation did not exhibit a significant mediating effect in the parental psychological control–NSSI association, depression emerged as a robust mediator in this relationship. Furthermore, paternal and maternal psychological control influenced NSSI through the following chain mediation pathway: paternal/maternal psychological control → relative deprivation → depression → NSSI.

Peer relationships moderated four key pathways: (a) paternal psychological control → NSSI, (b) paternal psychological control → depression, (c) relative deprivation → depression, and (d) depression → NSSI. However, this moderating pattern was absent in the maternal psychological control–NSSI association, where peer relationships only moderated the depression–NSSI link. These differential patterns highlight distinct gendered mechanisms underlying paternal versus maternal psychological control in influencing NSSI.

The study underscores the adverse developmental consequences of parental psychological control on college students and the protective capacity of peer relationships. These insights can inform campus mental health policies targeting family–peer system interactions and guide practitioners in designing multilevel interventions to reduce NSSI risk.

## Figures and Tables

**Table 1:** Descriptive statistics and correlations for the variables.

	1	2	3	4	5	6	7	8
1. Gender	1							
2. Age	−0.04	1						
3. PPC	−0.16 **	0.01	1					
4. MPC	−0.15 **	−0.02	0.77 ***	1				
5. RD	−0.09 **	−0.07 *	0.18 ***	0.21 ***	1			
6. DN	−0.09 **	−0.07 *	0.25 ***	0.31 ***	0.37 ***	1		
7. PR	0.08 **	0.01	−0.33 ***	−0.35 ***	−0.28 ***	−0.45 ***	1	
8. NSSI	−0.04	−0.10 **	0.12 ***	0.11 ***	0.13 ***	0.34 ***	−0.15 ***	1
M ± SD		19.26 ± 0.99	2.17 ± 1.02	1.95 ± 1.03	2.28 ± 1.02	1.55 ± 0.56	3.89 ± 0.94	0.10 ± 0.46

Note: 1 = gender; 2 = age; 3 = paternal psychological control, PPC; 4 = maternal psychological control, MPC; 5 = relative deprivation, RD; 6 = depression, DN; 7 = peer relationships, PR; 8 = non-suicidal self-injury, NSSI; * *p* < 0.05, ** *p* < 0.01, *** *p* < 0.001.

**Table 2:** Total, indirect, and mediated effects of paternal psychological control on NSSI.

	Effect	Boot SE	Boot LLCI	Boot ULCI	Ratio
PPC → RD → NSSI	−0.0001	0.002	−0.005	0.004	
PPC → DN → NSSI	0.023	0.005	0.013	0.035	47.91%
PPC → RD → DN → NSSI	0.007	0.002	0.004	0.011	14.58%
Total Indirect effect	0.03	0.006	0.018	0.044	62.49%
Direct effect	0.018	0.01	−0.005	0.04	37.5%
Total effect	0.048	0.01	0.02	0.07	100%

Note: PPC, paternal psychological control; RD, relative deprivation; DN, depression; NSSI, non-suicidal self-injury.

**Table 3:** Total, indirect, and mediated effects of maternal psychological control on NSSI.

	Effect	Boot SE	Boot LLCI	Boot ULCI	Ratio
MPC → RD → NSSI	0.0002	0.002	−0.005	0.005	
MPC → DN → NSSI	0.03	0.007	0.018	0.045	72.38%
MPC → RD → DN → NSSI	0.008	0.002	0.004	0.013	20.46%
Total Indirect effect	0.039	0.007	0.025	0.05	92.85%
Direct effect	0.003	0.01	−0.02	0.03	7.15%
Total effect	0.042	0.01	0.02	0.06	100%

Note: MPC, maternal psychological control; RD, relative deprivation; DN depression; NSSI, non-suicidal self-injury.

**Table 4:** Testing the moderated mediation effect of paternal psychological control on NSSI.

Variables	Model 1 (RD)			Model 2 (DN)			Model 3 (NSSI)		
	*β*	*t*	*95% CI*	*β*	*t*	*95% CI*	*β*	*t*	*95% CI*
Gender	−0.11	−1.95	[−0.22, 0.001]	−0.07	−1.46	[−0.17,0.02]	−0.004	−0.19	[−0.05, 0.04]
Age	−0.06	−2.37 *	[−0.12, −0.01]	−0.05	−2.18 *	[−0.17, −0.005]	−0.04	−3.22 **	[−0.06, −0.01]
PPC	0.09	3.21 **	[0.03, 0.15]	0.08	3.09 **	[0.03, 0.13]	0.02	1.85	[−0.001, 0.05]
PR	−0.25	−8.40 ***	[−0.31, −0.19]	−0.35	−12.98 ***	[−0.40, −0.30]	0.003	0.26	[−0.02, 0.03]
RD				0.23	8.71 ***	[0.18, 0.29]	0.001	0.05	[−0.02, 0.02]
DN							0.12	8.38 ***	[0.09, 0.14]
PPC × PR	0.04	1.75	[−0.005,0.10]	0.06	2.39 *	[0.01, 0.11]	−0.02	−2.0 *	[−0.04, −0.001]
RD × PR				−0.05	−2.16 *	[−0.09, −0.005]	−0.003	−0.30	[−0.02, 0.02]
DN × PR							−0.04	−3.66 ***	[−0.06, −0.02]
R^2^	0.10			0.28			0.14		
F	24.97 ***			63.67 ***			20.18 ***		

Note: PPC, paternal psychological control; RD, relative deprivation; DN, depression; PR, peer relationships. * *p* < 0.05, ** *p* < 0.01, *** *p* < 0.001.

**Table 5:** Testing the moderated mediation effect of maternal psychological control on NSSI.

Variables	Model 1 (RD)			Model 2 (DN)			Model 3 (NSSI)		
	*β*	*t*	*95% CI*	*β*	*t*	*95% CI*	*β*	*t*	*95% CI*
Gender	−0.10	−1.83	[−0.21, 0.007]	−0.06	−1.14	[−0.15, 0.04]	−0.01	−0.48	[−0.06, 0.03]
Age	−0.06	−2.27 *	[−0.12, −0.01]	−0.06	−2.09 *	[−0.10, −0.003]	−0.04	−3.18 **	[−0.06, −0.01]
MPC	0.12	4.10 ***	[0.06, 0.18]	0.14	5.05 ***	[0.08, 0.18]	0.004	0.32	[−0.02, 0.03]
PR	−0.24	−7.94 ***	[−0.30, −0.18]	−0.33	−12.15 ***	[−0.38, −0.28]	−0.004	−0.33	[−0.03, 0.02]
RD				0.23	8.58 ***	[0.18, 0.28]	0.0007	0.05	[−0.02, 0.02]
DN							0.12	8.33 ***	[0.09, 0.14]
MPC × PR	0.04	1.26	[−0.02, 0.09]	0.02	0.84	[−0.03, 0.07]	−0.01	−0.47	[−0.03, 0.02]
RD × PR				−0.04	−1.95	[−0.09,0.002]	−0.01	−0.45	[−0.02, 0.02]
DN × PR							−0.04	−3.47 ***	[−0.06, −0.02]
R2	0.10			0.28			0.13		
F	25.89 ***			65.71 ***			19.27 ***		

Note: MPC, maternal psychological control; RD, relative deprivation; DN, depression; PR, peer relationships. * *p* < 0.05, ** *p* < 0.01, *** *p* < 0.001.

## Data Availability

The datasets used and analyzed during the current study are available from the corresponding author on reasonable request.
